# Personalized Armored TCR-Redirected T Cell Therapy for Liver/Organ Transplant with Recurrent Cancer

**DOI:** 10.3390/cells10081861

**Published:** 2021-07-22

**Authors:** Morteza Hafezi, Anthony Tan, Antonio Bertoletti

**Affiliations:** 1 Emerging Infectious Diseases Program, Duke-NUS Medical School, Singapore 169857, Singapore; e0081747@u.nus.edu (M.H.); anthony.tan@duke-nus.edu.sg (A.T.); 2 Singapore Immunology Network, Singapore Agency for Science, Technology & Research (A*STAR), Singapore 169857, Singapore

**Keywords:** immunotherapy, tumor, organ transplant, immunosuppression

## Abstract

Hepatitis B virus-related hepatocellular carcinoma recurrence after liver transplantation (LT) is notoriously difficult to manage and fatal. As a therapeutic option, adoptive cell therapy with HBV-specific TCR-redirected T cells could be employed to target and control relapses in these patients. However, indispensable immunosuppressive medications post-transplantation can significantly hinder the optimum efficacy of such therapy in the clinic. Here we report a new class of Armored TCR T cells which are able to attack recurrent cancer cells in liver transplanted recipients, while temporarily evading immunosuppressant drugs. We believe this strategy could open up new opportunities for treating pathologies under immunosuppressant treatment.

Hepatocellular carcinoma (HCC), is the most common type of adult liver malignancy and a leading cause of cancer-related mortality globally. Different etiological factors are involved in HCC development, among which chronic Hepatitis B virus (HBV) infection accounts for approximately 50% of HCC cases globally [1]. Among all therapeutic modalities, liver transplantation (LT) remains the only curative treatment of choice for selected HBV-HCC patients [2]. However, 10–20% of liver transplanted recipients experience HBV-HCC recurrence within years post-LT, leaving them with limited therapeutic options. Post-transplant recurrence (PTR) typically occurs systemically and therefore it is fatal and difficult to manage [3]. In such scenario, most of the current therapeutic modalities are ineffective and long-term cure of PTR is rarely observed. As such, there is an unmet need for novel therapeutic approaches for treating liver transplant subjects with recurrent HBV-HCC.

Over the past decade, special attention was given to immune based therapy regimens such as immune check point inhibitor (ICI) and adoptive cell therapy as a promising treatment choice for HCC patients. ICIs are a type of immunotherapy drug which aims to rejuvenate and/or induce anti-tumor T cell responses. Clinical trials using ICIs have shown promising outcome in treating primary HCC [4]. However, this approach may put organ transplanted subjects at risk as it systemically activates the immune response which might lead to a wide range of immune-mediated adverse events (IMAEs) and unpredicted inflammation or even graft rejection. That is why at the moment, liver transplanted patients with HBV-HCC relapses are excluded from this therapeutic approach. In such scenario, targeted adoptive T cell therapy can represent an alternative immunotherapeutic option. In the case of HBV-HCC, cancer nodules express HBV-associated antigens which can be employed as a tumor-specific antigen to engineer HBV-redirected T cells. Engineered T cells equipped with chimeric antigen receptor (CAR) or classical T cell receptors (TCRs) against HBV-specific antigens detect and lyse HBV-infected HCC tumor cells [5,6]. Clinical trials utilizing genetically engineered HBV-redirected T cells illustrate the promise of this therapy in HBV-associated HCC. In the case of TCR T cells, our laboratory demonstrated that therapeutic HBV-redirected TCR T cells can be effectively employed for treatment of extrahepatic HBV-HCC relapses post transplantation [7,8]. Importantly, since human leukocyte antigen (HLA) matching is not carried out routinely for LT subjects, these autologous TCR-redirected T cells are able to exclusively lyse the HBV-HCC relapses and not the HBV-infected untransformed hepatocyte in the transplanted organ which provide another layer of safety for immunotherapy application in transplanted subjects.

## Engineering Armored Immunosuppressive Drug-Resistant T cells for Immunotherapy Application

**Figure 1:**
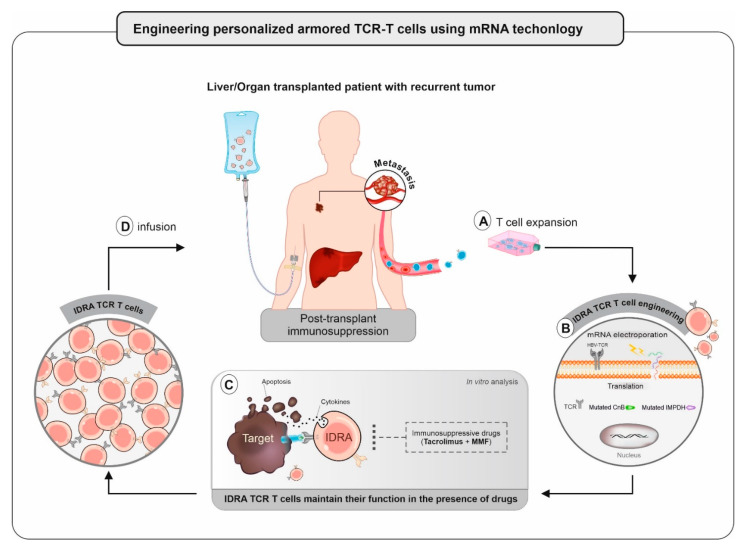
Schematic illustrating the development of IDRA TCR redirected T cells using mRNA technology. (**A**) Expansion and activation of autologous T cells for T cell editing; (**B**) concomitant mRNA electroporation of TCR and mutated forms of CnB, IMPDH transgenes in order to engineer IDRA TCR T cells; (**C**) edited IDRA TCR T cells are able to transiently evade immunosuppression and maintain their function in the presence of drugs using immune assays; (**D**) following quality control, autologous IDRA TCR T cells infused back into the transplanted subject.

Despite the encouraging outcome of utilizing T cell immunotherapy in the initial trials, various factors present in the patient sera might adversely affect the optimum efficacy of such treatment. One of the main obstacles for implementing immunotherapy in organ transplanted subjects is the post-transplantation immunosuppression (IS) regimens which might hinder the efficacy of T cell therapy in these patients [9]. In case of LT, maintenance IS typically started by Tacrolimus alone or in combination with Mycophenolate mofetil (MMF) post-transplantation continues lifelong. These drugs were designed to broadly suppress the T cell-mediated immune responses and prevent graft rejection. With regard to that, our recent study showed that even short exposure of these drugs has considerable impact on the function and migration of T cells in vitro and in vivo that appears to be correlated with clinical efficacy of T cell therapy [9]. Hence, solutions are required to maintain engineered T cells function while trafficking towards recurrent HBV-HCC nodules in the presence of obligate immunosuppression. Previous attempts showed that this can be achieved through genetic engineering using CRISPR/Cas9 [10] and other viral systems [11,12]. According to Brewin et al., stable expression of mutated versions of Tacrolimus-specific adapter protein markedly recovered T cell function in the presence of clinically relevant concentration of the Tacrolimus [11]. On the other hand, recent report by Amini et al. showed that this feature could be achieved by disrupting Peptidyl-prolyl cis-trans isomerase FKBP1A protein using ribonucleoprotein (RNP) complex electroporation [10]. While these strategies might be beneficial in the short term, but in the long run, stable gene editing might lead to uncontrolled proliferation and activation of the edited cells which may cause graft rejection. This must be taken into consideration, especially where large number of T cells are infused to each transplant recipient. As such for safety purposes, we took advantage of mRNA electroporation as an alternative strategy to develop safe and efficient Immunosuppressive Drug Resistant Armored (IDRA) HBV-redirected TCR T cells (Figure 1). To achieve this, mRNA encoding HBV-specific TCR, mutated forms of calcineurin subunit B (CnB) and inosine-5′-monophosphate dehydrogenase (IMPDH), both drug specific target proteins, were concomitantly electroporated to the autologous T-cells. Based on our finding, these edited T cells are able to function properly even in the face of clinically relevant concentration of IS regimens in vitro. Importantly, unlike stable gene editing, the mRNA strategy limits the functional lifespan of modified T cells to about 3–5 days in vivo, after which these T cells revert back to their native specificity and become drug-sensitive. Therefore, this strategy avoids the establishment of potentially IDRA alloreactive T cells in transplant subject, which markedly reduce the chance of potential organ rejection. In addition to the safety consideration above, the transient nature of protein expression mediated by mRNA also allows clinical trials to design and develop a therapy in a personalized manner through intra-patient dose-escalation protocols. As such, trials employing mRNA technology might treat more patients at optimal or near-optimal doses and avoid exposing many patients to subtherapeutic doses of TCR T cell therapy. At last, we demonstrated that this strategy is not only restricted to LT recipients with recurrent HBV-HCC but also can be applied as therapeutic approach for other viral pathologies that occur under obligate immunosuppression. Refractory cytomegalovirus (CMV) or Epstein–Barr virus (EBV) infection remain a major risk factor for mortality of transplant recipients. Although virus-specific T cell therapy showed clinical benefits for treating such patients in the clinic, but at the moment it is not frequently used due to inhibitory effect of immunosuppressive medications. Our demonstration showed how the IDRA feature could be successfully transferred to other specific TCR like EBV-redirected TCR T cells. The ability to engineer T-cells with such transient expression characteristics provides an advance in the field and solves a problem that is not addressed by current methods. In our opinion this strategy could markedly improve the current available immunotherapy and open up new therapeutic possibilities for the pathologies under indispensable immunosuppression. We are looking forward to seeing this new class of immunotherapy pass the clinical trials with success and come to the clinics in the near future.
